# Pemphigus Foliaceus after COVID-19 Vaccination: A Report of Two Cases

**DOI:** 10.1155/2023/1218388

**Published:** 2023-10-19

**Authors:** Nguyen Nhat Pham, Thuy Thi Phan Nguyen, Thao Thi Phuong Vu, Hao Trong Nguyen

**Affiliations:** Ho Chi Minh City Hospital of Dermato-Venereology, Ho Chi Minh City, Vietnam

## Abstract

Autoimmune bullous diseases (AIBDs) following coronavirus disease (COVID-19) vaccination have been previously documented in medical literature, given the comparable nature of the RNA antigen in these vaccines to that of the cellular nuclear matter. However, pemphigus foliaceus has been reported less frequently than other postimmunization AIBDs worldwide. Two women were admitted to our hospital with skin erosion over their faces, trunks, and extremities after receiving COVID-19 vaccination. Upon examination and consultation with pathologists, the diagnosis of pemphigus foliaceus was confirmed for both patients. In an effort to contribute to the knowledge on this intriguing topic, we present these two aforementioned cases of pemphigus foliaceus following COVID-19 vaccination, which may initially appear as a typical occurrence but exhibit some noteworthy characteristics.

## 1. Introduction

Autoimmune bullous disease (AIBD) is characterized by vesicles, bullous lesions, and/or mucosal erosions. Autoantibodies target epidermal keratinocyte anchoring factors such as desmosomes and hemidesmosomes, resulting in the disease. AIBD can be intraepidermal or subepidermal based on clinical appearance, histology, and immunofluorescence. Pemphigus (intraepidermal bullae subtype) is less common than subepidermal bullae, with an incidence of 0.6–32/million/year [1]. Vaccines are central in the fight against COVID-19, and since their introduction, their efficacy and adverse effects have been researched globally. AIBDs have been reported after COVID-19 vaccination, whereas pemphigus foliaceus is rarely documented. We present two cases of pemphigus foliaceus following COVID-19 vaccination in previously healthy patients.

## 2. Case Report

### 2.1. Case 1

**Figure 1:**
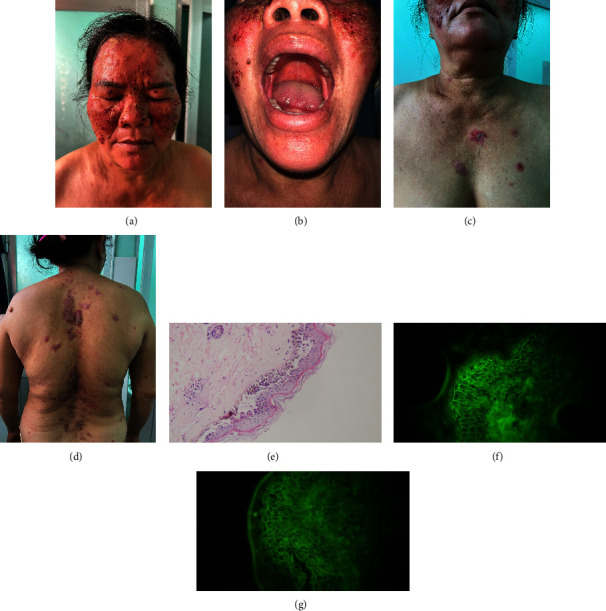
Patient 1 had many small-to-large erosions on her face (a) and trunk (c, d), without any mucosal lesions (b). Histopathological examination shows acantholysis above the stratum basalis (e). Direct immunofluorescence showed reticular intracellular C3 (f) and IgG (g) levels.

A 53-year-old woman was hospitalized for complaints of skin erosion that developed rapidly within 1 month (Figure 1). She had no family history of autoimmune diseases. The lesions first appeared on her face 3 weeks after the 4^th^ COVID-19 vaccination, which was the first AZD1222 (viral vector vaccine, AstraZeneca, UK) dose after three previous doses of BBIBP-CorV (inactivated vaccine, Sinopharm, China). Despite the local hospital diagnosis of pemphigus, and the treatment of 16 mg/day methylprednisolone for 2 weeks, the lesion spread throughout her trunk and extremities. She had a history of well-controlled hypertension with amlodipine 5 mg/day for approximately 2 years, without other health problems or treatment. She had multiple clear fluid-oozing erosions on her scalp, face, and back, with individual lesions measuring up to 10 cm^2^. Several erosions had thick crusts, and Nikolsky's sign was positive. Complete blood counts, serum creatinine, liver enzyme levels, and metabolic profiles were normal. Histopathology revealed acantholysis above the stratum basalis and lymphocyte and neutrophil infiltration in the dermis. Direct immunofluorescence (DIF) revealed IgG and C3 epidermal reticular deposition and absence of IgM, IgA, and fibrinogen. The diagnosis of pemphigus foliaceus was made based on the aforementioned findings. Oral methylprednisolone therapy equivalent to 1 mg/kg of prednisone was initiated daily; however, the patient did not respond well after 10 days of treatment, leading to further lesions. Rituximab was then started, and the patient was discharged a week later after she improved and the lesion partially healed. Two weeks later, the clinical response was excellent and the corticosteroid dose was reduced to 10 mg/day.

### 2.2. Case 2

**Figure 2:**
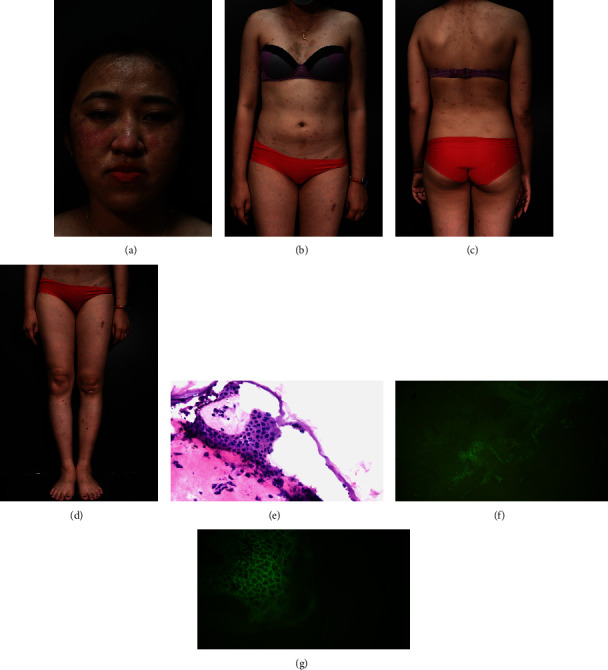
Patient 2 had scaling and oozing erosions on her face (a), trunk (b, c), and legs (d). Histopathological examination showed intraepidermal bullae formation (e). DIF showed net-like intracellular C3 (f) and IgG (g) levels.

A 30-year-old woman presented to the clinic with crusty erosion on her face. Her condition had fluctuated, with deteriorations and improvements, over the previous 4 months. No family history of autoimmune diseases was recorded. All symptoms started 2 months after her second dose of mRNA-1273 vaccine (Moderna, USA), with erosion and crusting on her neck and around the injection site, which spread to other places on her body (Figure 2). The patient was self-treated, with no improvement. On visiting the local hospital, she was diagnosed with atopic dermatitis and treated with methylprednisolone at 8 mg/day (highest dose). The lesions on her neck progressively healed; however, new blisters appeared on her face and torso. When the patient visited our hospital, she had crusting erosions on both cheeks and less erosion on her trunk with positive Nikolsky's sign. The blood test results were at normal levels. Histopathologically, acantholysis was observed above the stratum basalis. DIF showed intraepidermal net-like deposition of IgG and C3. These signs, symptoms, and laboratory results led to a diagnosis of pemphigus foliaceus. The patient responded to high-dose systemic corticosteroid equivalent to 1 mg/kg of prednisone combined with topical clobetasol propionate 0.05%. Her condition was stable for a month before the systemic corticosteroid was progressively reduced and the topical agent was changed to tacrolimus 0.1%. After 4 months, the patient responded well with the oral methylprednisolone dose equivalent to 10 mg/day prednisone and the lesions were healed.

## 3. Discussion

AIBDs are caused by autoimmunity to skin structures. Desmogleins 1 and 3, which act as desmosome adhesion molecules, are targets of IgG autoantibodies that characterize the autoimmunity of the pemphigus spectrum. T and B lymphocytes are crucial in the pathogenesis of these diseases [2]. It is unclear how autoimmune diseases develop after antiviral vaccination. However, because they may be exacerbated by external factors, including infections and medications, cross-reactivity across vaccine antigens, including adjuvants, may cause postvaccination autoimmunity. Studies have suggested that COVID-19 vaccinations may induce autoimmune diseases because they contain the genetic material of the virus [3]. Moreover, molecular mimicry and cascades of immunological events targeting the nuclear components of predisposed individuals can trigger the autoimmunity in autoimmune diseases. Therefore, the pathogenesis of AIBDs following COVID-19 immunization is predicted to follow the same immunological pattern.

Tissue-specific expression patterns of desmoglein isoforms and the antidesmoglein IgG profile can be used to explain the location of blister formation. Clinical, histological, and immunochemical investigations are used to diagnose and confirm AIBD [2]. A recent article summarized several cases of pemphigus after COVID-19 vaccination [4]. At the time of publication, pemphigus foliaceus appeared to be less prevalent than pemphigus vulgaris, with only one reported case. However, at the time of our report, at least six more cases of pemphigus foliaceus had been reported globally (Table 1) [5–9]. These case reports, which came from several nations and varied in their underlying diseases and immunization schedules, involved healthy individuals with new-onset foliaceus pemphigus. These characteristics are similar to those of our two cases.

Most cases in the literature had similar regimens with the BNT162b2 vaccine, which made our two cases unique. Our first patient had mixed regimens, three shots of BBIBP-CorV, and one AZD1222 dose, and the second patient was fully immunized with mRNA-1273. We did not find any patients with pemphigus foliaceus after mRNA-1273 and AZD 1222 vaccination. However, a report from Iran is notable as it is possibly the only documented case of pemphigus foliaceus associated with BBIBP-CorV vaccination [5]. Our first case was also related to BBIBP-CorV vaccine; however, the adverse effect manifested after the booster dose of AZD 1222. This made it challenging to identify whether the disease was induced by AZD 1222 or BBIBP-CorV. In addition, no case report involved both vaccines.

Another distinctive characteristic is the number of vaccine doses administered before the onset of pemphigus foliaceus. Similar to other earlier cases of the disease, our second patient began to manifest symptoms after receiving two mRNA-1273 doses. However, in our first patient, the onset of pemphigus foliaceus followed three doses of BBIBP-CorV and one dose of AZD 1222. We also used the Najaro scale to assess the possibility of vaccines contributing to the induction of this disease, similar to the study by Lua et al. [6, 10]. The vaccinations were identified as probable causes of pemphigus foliaceus in the first and second patients, respectively, with Najaro scores of 3 and 7. As we currently lack an effective method for identifying the role of medication when drug-induced allergic reactions occur, apart from the questionnaire-based inquiries outlined above, identifying the medicine that causes adverse effects in these case reports is challenging.

The time to the onset of vaccine-induced pemphigus foliaceus following the final vaccine dose ranged from 2 days to 2 months, as observed in the case studies cited earlier [5–9]. This was comparable to the latency time of our second case; however, we found no study on the latency of mRNA-1273 vaccine-induced pemphigus foliaceus. Determining the latency time in our first case was as difficult as identifying the vaccination that caused the condition. Based on the 3-week latency period, we hypothesize that AZD 1222 caused the disease because a much longer latency time of 5 months following the last BBIBP-CorV dose appeared inappropriate. However, we cannot determine which of the two vaccines caused pemphigus foliaceus due to the lack of adequate evidence.

Although most patients with pemphigus foliaceus reported worldwide were aged 50–83 years, our second patient was 30 years old [5–9]. Polydrug usage is widespread among older adults, compounding the difficulty in identifying the real cause of pemphigus foliaceus. Our first patient had taken other medications for up to 2 years, with no adverse effects. Moreover, she continued when she was admitted to the hospital without worsening disease. Therefore, we can exclude other medications as the origin of her condition.

We observed that almost all documented cases worldwide had comparable clinical features that were typical of pemphigus foliaceus [5–9]. Our two cases were also clinically typical since there were no mucosal lesions, and erosion was more prominent than bullous. Histopathology and DIF were performed for all case reports. However, indirect immunofluorescence was not performed in our cases as noted in two other studies [5, 8]. Corrá et al. performed indirect immunofluorescence, with negative results [7]. We considered the clinical and pathologic features of the patients to diagnose pemphigus foliaceus, which was consistent with other cases worldwide.

The majority of COVID-19-vaccine-induced pemphigus foliaceus patients improved after a short therapy, typically 2–4 weeks [5–9]. Corticosteroids (oral and topical) were frequently used in conjunction with azathioprine, mycophenolate mofetil, and rituximab. The first patient showed a quick response to rituximab, and the lesions healed within 3 weeks. The second patient responded well to oral and topical corticosteroid therapy after 4 weeks. Based on our experience and the literature, COVID-19-vaccine-induced pemphigus foliaceus responds to treatment better than classical cases. However, owing to the scarcity of pemphigus foliaceus cases in general, and postvaccination in particular, studies to confirm our assumption would be challenging to conduct.

In conclusion, based on reports worldwide, AIBDs and foliaceus pemphigus following COVID-19 immunization should receive greater attention. Rare cases of pemphigus foliaceus after COVID-19 vaccination are not difficult to diagnose because of the typical clinical manifestations, histopathology, and immunofluorescence results. However, lack of previous studies and other factors make it difficult to determine which vaccine causes the disease. The disease may appear serious; however, based on our experience and the literature, it responds well to appropriate treatment.

## Figures and Tables

**Table 1:** Known case report(s) of pemphigus foliaceus after COVID-19 vaccination (including our 2 cases).

Authors (country)	Num. of case (s)	Age/sex of patient (s)	Vaccine regimen	Onset milestones	Concomitant drugs	Histopathology (DIF)	Treatment (s) (response)
Our cases (Vietnam)	2	53/female	Mixed 1 AZD1222 dose following 3 BBIBP-CorV doses	3 weeks following 4^th^ AZD1222 dose in the mixed regimen	Amlodipine	Acantholysis above the stratum basalis, dermal lymphocyte, and neutrophil infiltration (intraepidermal IgG and C3)	Corticosteroid, rituximab (almost complete response in 3 weeks)
		30/female	2 doses of mRNA-1273	2 months following 2^nd^ mRNA-1273 dose	None	Acantholysis above the stratum basalis (intraepidermal IgG and C3)	Topical and systemic corticosteroid (almost complete response in 4 weeks)

Pourani et al. [5] (Iran)	1	75/male	3 doses of BBIBP-CorV	2 weeks after 3^rd^ dose	None	Superficial epidermal bullae, mild spongiosis, superficial dermal perivascular inflammation (intraepidermal IgG and C3)	Topical corticosteroid, rituximab (significant response in 4 weeks)

Lua et al. [6] (Singapore)	1	83/male	2 doses of BNT162b2	2 days after 2^nd^ dose	N/A	Subacute spongiotic dermatitis (C3 dermal-epidermal junction and intercellular deposition)	Prednisolone (good clinical response)

Corrá et al. [7] (Italy)	2	80/male	3 doses of BNT162b2	17 days after 3rd dose	Amiloride, hydrochlorothiazide, esomeprazole	Subcorneal acantholysis with neutrophilic infiltration within the blister (PT1: negative; PT2: intercellular IgG deposits)	Oral corticosteroid, rituximab, MMF (probably good clinical response)
		66/female	2 doses of BNT162b2	4 weeks after 2nd dose	Rabeprazole, ticlopidine, atorvastatin, amlodipine, hydrochlorothiazide		

Hali et al. [8] (Morocco)	1	50/female	2 doses of BNT162b2	15 days after 2^nd^ dose	None	Superficial epidermal blistering process, intact basal layer, intraepidermal eosinophils (intracellular IgG and C3)	Oral corticosteroid (complete response in 3 weeks)

Yıldırıcı et al. [9] (Turkey)	1	65/male	2 doses of BNT162b2 (6 weeks apart)	1 months after 1^st^ dose; 2 weeks after 2^nd^ dose	Nebivolol, valsartan‐hydrochlorothiazide	Intraepidermal acantholytic blister, abundant neutrophils, and scarce eosinophils (intercellular IgG and C3)	Oral corticosteroid, azathioprine (marked response in 2 weeks)

## Data Availability

No data were used to support the findings of this study.
